# Anti-Inflammatory, Antioxidant, and Reparative Effects of *Casearia sylvestris* Leaf Derivatives on Periodontium In Vitro

**DOI:** 10.3390/antiox14080901

**Published:** 2025-07-23

**Authors:** Angélica L. R. Pavanelli, Maria Eduarda S. Lopes, André T. Reis, Flávio A. Carvalho, Sven Zalewski, André G. dos Santos, Joni A. Cirelli, James Deschner, Andressa V. B. Nogueira

**Affiliations:** 1 Department of Periodontology and Operative Dentistry, University Medical Center of the Johannes Gutenberg University, 55131 Mainz, Germany; angelica.pavanelli@unesp.br (A.L.R.P.); james.deschner@uni-mainz.de (J.D.); 2 Department of Diagnosis and Surgery, School of Dentistry at Araraquara, São Paulo State University (UNESP), Araraquara 14801903, Brazil; maria.e.lopes@unesp.br (M.E.S.L.); joni.cirelli@unesp.br (J.A.C.); 3 Department of Drugs and Medicines, School of Pharmaceutical Sciences, São Paulo State University (UNESP), Araraquara 14800903, Brazil; at.reis@unesp.br (A.T.R.); flavio.a.carvalho@unesp.br (F.A.C.); sven.zalewski@unesp.br (S.Z.); andre.gonzaga@unesp.br (A.G.d.S.)

**Keywords:** periodontal diseases, *Casearia sylvestris*, anti-inflammatory, antioxidant, wound healing

## Abstract

Gingival inflammation compromises the integrity of the gingival epithelium and the underlying tissues, highlighting the need for adjuvant therapies with immunomodulatory and healing properties. *Casearia sylvestris*, a medicinal plant known as guaçatonga, is traditionally used to treat inflammatory lesions. This study aimed to investigate the effects of *C. sylvestris* on the synthesis of pro- and anti-inflammatory, proteolytic, and antioxidant molecules and on wound healing in epithelial cells. A human telomerase-immortalized gingival keratinocyte cell line (TIGKs) was used, and cells were exposed to *Escherichia coli* lipopolysaccharide (LPS) in the presence and absence of *C. sylvestris* extract, its diterpene-concentrated fraction, and its clerodane diterpene casearin J for 24 h and 48 h. Gene expression and protein synthesis were analyzed by RT-qPCR and ELISA, respectively. Nitric oxide (NO) and NF-κB activation were analyzed by Griess reaction and immunofluorescence, respectively. Additionally, cell viability was evaluated by alamarBlue^®^ assay, and an automated scratch assay was used for wound healing. LPS significantly increased the expression of cytokines (TNF-α, IL-1β, IL-6, IL-8, IL-10, IL-17), proteases (MMP-1 and MMP-13), iNOS as well as NO synthesis, and triggered NF-κB nuclear translocation. It also reduced IL-4 expression, cell viability, and cellular wound repopulation. Treatment with *C. sylvestris* derivatives significantly abrogated all aforementioned LPS-induced effects by 80–100%. Furthermore, even at higher concentrations, *C. sylvestris* did not affect cell viability, thus proving the safety of its derivatives. *C. sylvestris* exerts anti-inflammatory, antiproteolytic, and antioxidant effects on gingival keratinocytes, highlighting its potential as a valuable adjunct in the prevention and treatment of periodontal diseases.

## 1. Introduction

The oral mucosa is lined with stratified squamous epithelium that plays essential roles in maintaining oral homeostasis. Oral epithelial cells act as a physical barrier against pathogenic microorganisms and actively participate in the innate immune response by producing cytokines and chemokines that modulate inflammation and tissue repair. Moreover, these cells interact with the oral microbiota, contributing to the balance between health and disease in the oral cavity [1,2].

Gingivitis is a reversible inflammation of the gingival tissues caused primarily by the accumulation of dental biofilm, which is a complex community of microorganisms adhering to the tooth surface, triggering a host inflammatory response. This condition is characterized by erythema, edema, and bleeding on probing, without loss of attachment or destruction of the alveolar bone [3]. If left untreated, gingivitis can progress to periodontitis, leading to irreversible damage to the periodontal tissue [4,5]. During gingivitis, there is an increase in the cellular production of pro-inflammatory cytokines, such as interleukin (IL)-1β, tumor necrosis factor-alpha (TNF-α), and IL-6, which contribute to tissue destruction and disease progression [4,5,6,7]. In addition, activation of the IL-23/IL-17 axis also plays a critical role in the pathogenesis of periodontitis, a more advanced stage of periodontal disease [8].

Proper control of the oral microbiota is essential for preventing chronic inflammation and destruction of periodontal tissues. However, excessively prolonged inflammation can disturb microbial balance, leading to dysbiosis and intensification of immune responses [9,10]. Therefore, therapeutic strategies should focus on modulating the microbiota while preserving homeostasis and preventing damage to periodontal tissues. Conventional periodontal treatment includes mechanical removal of biofilm by manual and/or ultrasonic instrumentation, along with proper oral hygiene instructions. These approaches aim to reduce microbial load and inflammation, promoting tissue regeneration. However, the search for adjunctive therapies that enhance conventional treatments has led to investigations of natural compounds with anti-inflammatory and antimicrobial properties [11,12,13].

In recent years, there has been growing interest in the use of natural polymers and plant-based compounds as adjuvant therapies in maintaining or restoring oral health. The concept of “green dentistry” advocates environmentally friendly and biologically safe alternatives for oral health maintenance. Natural biopolymers such as chitosan and plant-derived extracts, including propolis and cranberry, have shown promising anti-inflammatory, antimicrobial, and tissue regenerative properties [14]. The incorporation of herbal agents into oral care formulations reflects a growing trend towards the development of sustainable and biocompatible therapeutic strategies.

Several medicinal plants have been studied for their benefits in periodontal health. For example, garlic (*Allium sativum*) contains bioactive compounds with antimicrobial activity against periodontal pathogens, such as *Porphyromonas gingivalis* [15]. Other plants, such as green tea (*Camellia sinensis*), turmeric (*Curcuma longa*), and passion fruit (*Passiflora edulis*) bagasse extract have also shown anti-inflammatory and antioxidant properties that may help manage periodontal diseases [16,17,18]. *Casearia sylvestris* Swartz (Salicaceae), popularly known as guaçatonga in Brazil, is a Latin American medicinal plant traditionally used for its anti-inflammatory, wound healing, antimicrobial, and antiulcer properties [19,20,21,22]. *C. sylvestris* has been used in formulations to treat cutaneous lesions, and infusions of its leaves have been used to combat gastric ulcers [19,21]. In vitro studies have demonstrated that the ethanolic extract of *C. sylvestris* leaves exhibit antimicrobial activity against oral pathogens, suggesting its potential use as a therapeutic agent in oral infections, including periodontal diseases [23,24]. This pharmacological potential is primarily attributed to its rich content of secondary metabolites, including flavonoids and a particular group of clerodane-type diterpenes that are structurally similar to casearin and are collectively known as casearin-like diterpenes [25,26]. These compounds are the main bioactive constituents of *C. sylvestris* and are associated with anti-inflammatory, antioxidant, and cytoprotective properties [19]. In addition to the crude ethanolic extract, the present study therefore incorporated a diterpene-enriched fraction and the isolated compound casearin J to delineate the contributions of these diterpenoids. Therefore, the aim of this in vitro study was to investigate whether *C. sylvestris* derivatives can modulate the synthesis of pro- and anti-inflammatory, proteolytic, and antioxidant molecules and wound healing in gingival keratinocytes.

## 2. Materials and Methods

### 2.1. Cell Culture

A human telomerase immortalized gingival keratinocyte cell line (TIGKs, CRL-3397, ATCC, Manassas, VA, USA) was used. Cells were cultured in Keratinocyte Growth Medium (KGM-Gold; Lonza, Basel, Switzerland) at 37 °C and 5% CO_2_. Gingival keratinocytes were seeded in 12-well plates (80,000 cells/well) and grown until reaching 70–80% confluence. The culture medium was changed every two days. In order to simulate an inflammatory environment in vitro, cells were exposed to 1 µg/mL of LPS from *E. coli* (Sigma-Aldrich, St. Louis, MO, USA). After 30 min, cells were treated with 1 µg/mL of *C. sylvestris* ethanolic extract (extract), 1 µg/mL of *C. sylvestris* fraction (F2), and 50 µg/mL of its clerodane diterpene casearin J (Cas J). Although a higher dose of Cas J (50 µg/mL) was used compared to the ethanolic extract or fraction F2 (1 µg/mL), this concentration was selected based on the absence of cytotoxicity in keratinocytes and aimed to ensure observable biological effects in the in vitro model. Untreated cells were used as control.

### 2.2. Plant Material, Extraction, Purification, and Quantification of Cas J

Leaves were collected from 10 specimens of *C. sylvestris* at the School of Pharmaceutical Sciences, São Paulo State University, Araraquara, SP, Brazil. Furthermore, this study was registered in the Brazilian National System for the Management of Genetic Heritage (SisGen) under the code A9B40A7. Leaves were dried at 40 °C for 3 d and powdered using a knife mill. The dried and powdered leaves (3 kg) were extracted by maceration with ethanol (3:15 *w*/*v*, 72 h) at 40 °C. Ethanol was evaporated in an IKA DEST KV 05S3 evaporator to yield the dry extract (300 g, 10.0% *w*/*w*). The extract (62 g) was submitted to solid-phase extraction in a glass column (20 × 10 cm) using silica gel (63–200 μm)/activated charcoal (1:1, *w*/*w*) and eluted with hexane–ethyl acetate 95:05 (fraction 1—F1), ethyl acetate (fraction 2—F2), and methanol (fraction 3—F3). F2 (12.6 g) was submitted to silica gel column chromatography (20 × 5 cm, 40–63 μm) eluted with 1.3 L of hexane–ethyl acetate–isopropanol gradient (85:14:01; 78:20.5:1.5; 76:22.4:1.6; 73:25.2:1.8; 68:29.9:2.1, and 60:37.2:2.8), resulting in 38 subfractions. Cas J was purified from subfractions 11–12 by preparative HPLC-PDA/UV (Shimadzu^®^ Corporation, Kyoto, Japan) with an Agilent Eclipse XDB HPLC-PDA/UV C18 column (250 × 21.2 mm, 7 μm) using methanol 65% for 30 min, 15 mL/min flow rate, 1.8 mL injection volume, and monitored at 235 nm to yield 84.0 mg of Cas J (t R: 14.1 min; 98.3% purity). Cas J was identified by spectrometric data and quantified in extract and F2 according to Carvalho et al. (2018) [27]. The Cas J content in extract and fraction F2 was 0.2 and 3.9%, respectively, while the total casearin content in extract and fraction F2 was 11.9 and 58.4%, respectively.

### 2.3. Real-Time PCR

Total RNA was extracted using the RNeasy Protect Mini Kit (Qiagen, Hilden, Germany). RNA concentration was determined by spectrophotometry (NanoDrop ND-2000, Thermo Fisher Scientific, Waltham, MA, USA). Reverse transcription of 500 ng of RNA into cDNA was performed using the iScript™ Select cDNA Synthesis Kit (Bio-Rad Laboratories, Munich, Germany), following the manufacturer’s protocol. PCR reactions were conducted using SYBR Green PCR Master Mix (SsoAdvanced Universal SYBR Green Supermix, Bio-Rad Laboratories) and specific primers (QuantiTect Primer Assay, Qiagen). Gene expression of cytokines, including *IL-1β*, *IL-4*, *IL-6*, *IL-8*, *IL-10*, *IL-17*, and *TNF-α*, *MMP-1* and *MMP-13*, and *iNOS*, was analyzed. Glyceraldehyde-3-phosphate dehydrogenase (*GAPDH*) was used as a reference gene. The PCR protocol included 1 μL of cDNA, 12.5 μL of SYBR Green, 2.5 μL of primers, and 9 μL of nuclease-free water. Initial heating was at 95 °C for 5 min, followed by 40 cycles of denaturation at 95 °C for 10 s, and annealing/extension at 60 °C for 30 s. Data analysis was performed using the comparative threshold cycle (Ct) method.

### 2.4. ELISA

Quantification of TNF-α, IL-8, and MMP-1 protein levels in cell supernatants was performed using ELISA commercial kits (DuoSet Human ELISA Kit, R&D Systems, Minneapolis, MN, USA), according to the manufacturer’s instructions. Optical density was measured at 450 nm using a multimode microplate reader (BioTek Synergy H1, Agilent, Santa Clara, CA, USA). Optical correction was performed by subtracting readings at 540 nm, as recommended by the company. Cell counting was performed, and no significant differences between groups were observed.

### 2.5. Nitrite Analysis

Gingival keratinocytes were treated as previously described. Afterwards, the supernatants were collected, and the nitrite concentration was measured using the Griess Reagent Kit (MAK367, Sigma-Aldrich), following the manufacturer’s instructions. Supernatants were placed on a 96-well plate, and 10 µL of each Griess Reagent (I and II) were added. The volume was adjusted to 100 µL with Nitrite Assay Buffer. After incubation at room temperature for 10 min, the absorbance was measured at 540 nm using a microplate reader (BioTek Synergy H1, Agilent). Culture medium was used as a negative control. Nitrite concentration was determined from a nitrite standard curve. The experiment was performed in duplicate and repeated three times.

### 2.6. Immunofluorescence

Gingival keratinocytes were cultured in 24-well plates and, after 24 h, treated as previously described for up to 60 min. Cells were then washed with phosphate-buffered saline, fixed with 4% paraformaldehyde (Sigma-Aldrich) for 10 min, and permeabilized with 0.1% Triton X-100 (Sigma-Aldrich) for 5 min. Subsequently, cells were blocked with skimmed milk for 1 h. After washing, cells were incubated with a rabbit primary antibody against NF-κB p65 (1:400, D14E12, Cell Signaling Technology, Danvers, MA, USA) for 90 min. Then, cells were incubated with a goat anti-rabbit IgG CY3-conjugated secondary antibody (1:2000, ab6939, Abcam, Cambridge, MA, USA) for 45 min. Nuclear translocation of NF-κB p65 was observed by fluorescence using the ZOE™ Fluorescent Cell Imager (Bio-Rad Laboratories). Untreated cells served as control. The experiment was repeated three times.

### 2.7. Cell Viability

Cell viability was assessed using the alamarBlue assay (Invitrogen, Karlsruhe, Germany), following the manufacturer’s instructions. This method uses resazurin as a cell health indicator by measuring its reduction to resorufin. Briefly, gingival keratinocytes were seeded in 24-well plates (5000 cells/well) and cultured for 24 h, followed by treatment as previously described. Untreated cells served as control. After 24 h and 48 h of treatment, the medium was replaced with fresh medium containing 10% alamarBlue and incubated at 37 °C for 4 h. Then, 100 µL of the solubilization solution was transferred to 96-well plates, and absorbance was measured at 570 nm and 600 nm using a multimode reader (BioTek Synergy H1, Agilent). Results were expressed as the percentage difference in alamarBlue reduction between groups, according to the manufacturer’s protocol. The experiment was performed in duplicate and repeated three times.

### 2.8. Wound Healing

An in vitro wound healing model was used as previously published [18]. To evaluate the effects of *C. sylvestris* compounds on gingival keratinocytes, cells were seeded in 24-well plates (110,000 cells/well) and maintained until reaching confluence over 24 h. Subsequently, a uniform scratch was made in each well of the monolayer cells using the AutoScratch Wound Making Tool (Agilent, Santa Clara, CA, USA), according to the manufacturer’s instructions. After wound formation, wells were washed three times with culture medium to remove non-adherent cells and debris. Then, cells were treated as described above. Untreated cells served as control. High-resolution kinetic images were captured every two hours over 72 h, using the Lionheart FX Automated Microscope (Agilent). Wound healing analysis was performed using the Scratch Assay App (Agilent), providing detailed reports on wound width, cellular wound repopulation, and cellular wound repopulation rate. The experiment was repeated three times.

### 2.9. Statistical Analysis

Statistical analysis was performed using GraphPad Prism software (version 9.5.0, GraphPad Software, San Diego, CA, USA). Data were expressed as mean ± standard error of the mean (SEM). Data normality was assessed. For multiple comparisons, ANOVA followed by Dunnett’s test was used. A *p*-value < 0.0001 was considered statistically significant in all experiments.

## 3. Results

### 3.1. Regulatory Effects of C. Sylvestris Derivatives on Gene and Protein Expressions

**Figure 1:**
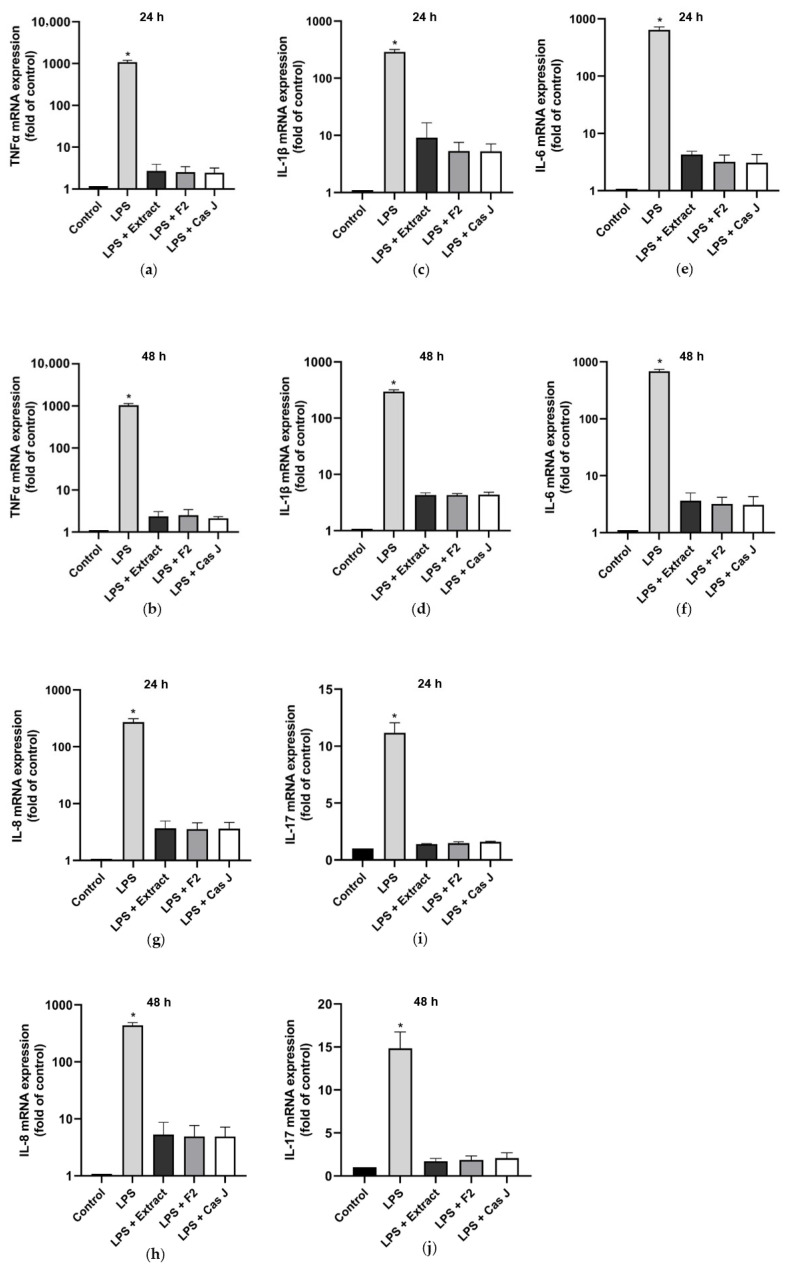
Regulation of the gene expressions of the pro-inflammatory cytokines *TNF-α* (**a**,**b**), *IL-1β* (**c**,**d**), *IL-6* (**e**,**f**), *IL-8* (**g**,**h**), and *IL-17* (**i**,**j**) in gingival keratinocytes exposed to LPS in the presence and absence of *C. sylvestris* derivatives after 24 h and 48 h, respectively. Untreated cells were used as a control. Values are expressed as mean ± SEM (*n* = 9). Log10 scale (**a**–**h**). * Significant (*p* < 0.0001) difference compared to all other groups. LPS (*E. coli* lipopolysaccharide), Extract (*C. sylvestris* ethanolic extract), F2 (*C. sylvestris* diterpene-concentrated fraction), Cas J (*C. sylvestris* clerodane diterpene casearin J).

First, we evaluated the gene expression of pro- and anti-inflammatory cytokines in gingival keratinocytes exposed to lipopolysaccharide (LPS) in the presence and absence of *C. sylvestris* extract (extract), its diterpene-concentrated fraction (F2), or its clerodane diterpene casearin J (Cas J) at 24 h and 48 h. Cells treated with LPS alone showed significantly (*p* < 0.0001) increased expression of *TNF-α*, *IL-1β, IL-6*, *IL-8*, and *IL-17* at 24 h and 48 h. However, *C. sylvestris* derivatives could significantly (*p* < 0.0001) counteract the stimulating effects of LPS on these pro-inflammatory molecules by 80–100% (Figure 1a–j, Table S1).

**Figure 2:**
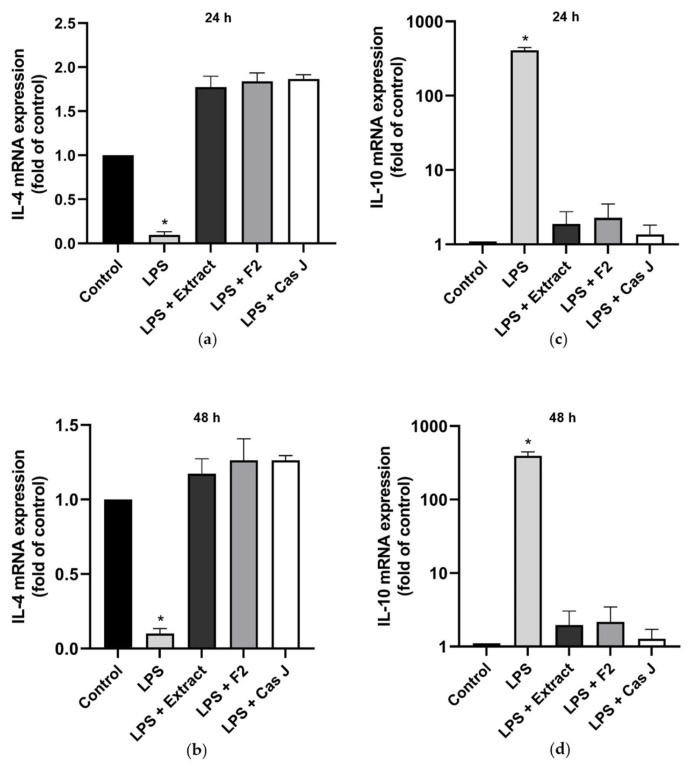
Regulation of the gene expressions of the anti-inflammatory cytokines *IL-4* (**a**,**b**) and *IL-10* (**c**,**d**) in gingival keratinocytes exposed to LPS in the presence and absence of *C. sylvestris* derivatives after 24 h and 48 h. Untreated cells were used as a control. Values are expressed as mean ± SEM (*n* = 9). Log10 scale (**c**,**d**). * Significant (*p* < 0.0001) difference compared to all other groups. LPS (*E. coli* lipopolysaccharide), Extract (*C. sylvestris* ethanolic extract), F2 (*C. sylvestris* diterpene-concentrated fraction), Cas J (*C. sylvestris* clerodane diterpene casearin J).

When cells were exposed to LPS, the constitutive *IL-4* gene expression was significantly (*p* < 0.0001) reduced (Figure 2a,b). However, when cells were simultaneously incubated with *C. sylvestris* extract, F2, or Cas J, this LPS-induced downregulation was completely abrogated (Figure 2a,b). In contrast to *IL-4*, the expression of *IL-10* was significantly enhanced in the presence of LPS. Nevertheless, this *IL-10* upregulation by LPS was significantly (*p* < 0.0001) inhibited by *C. sylvestris* extract, F2, or Cas J (Figure 2c,d, Table S1).

**Figure 3:**
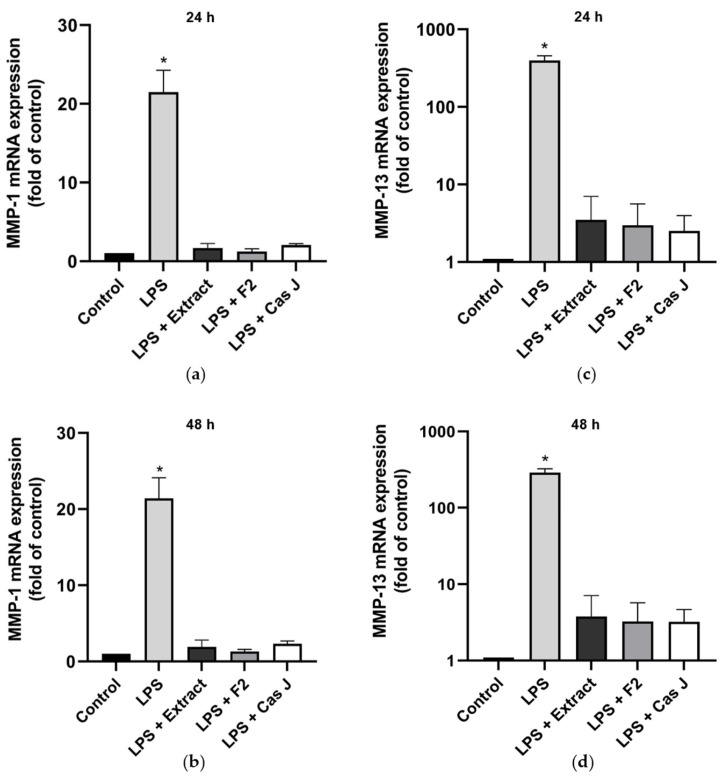
Regulation of the gene expressions of the proteolytic markers *MMP-1* (**a**,**b**) and *MMP-13* (**c**,**d**) in gingival keratinocytes exposed to LPS in the presence and absence of *C. sylvestris* derivatives after 24 h and 48 h. Untreated cells were used as a control. Values are expressed as mean ± SEM (*n* = 9). Log10 scale (**c**,**d**). * Significant (*p* < 0.0001) difference compared to all other groups. LPS (*E. coli* lipopolysaccharide), Extract (*C. sylvestris* ethanolic extract), F2 (*C. sylvestris* diterpene-concentrated fraction), Cas J (*C. sylvestris* clerodane diterpene casearin J).

Furthermore, we studied the effects of *C. sylvestris* derivatives on molecules involved in periodontal tissue degradation. LPS caused a significant upregulation of matrix metalloproteinases 1 (*MMP-1*) and 13 (*MMP-13*) as compared to control. When the cells were simultaneously exposed to *C. sylvestris* derivatives, the stimulating effect of LPS was significantly (*p* < 0.0001) diminished (Figure 3a–d, Table S1).

**Figure 4:**
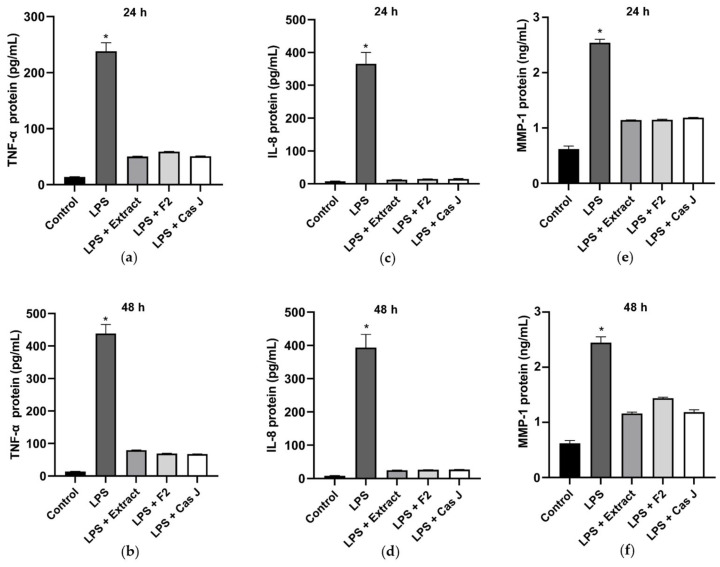
Protein levels of TNF-α (**a**,**b**), IL-8 (**c**,**d**), and MMP-1 (**e**,**f**) in supernatants of gingival keratinocytes exposed to LPS in the presence and absence of *C. sylvestris* derivatives after 24 h and 48 h. Untreated cells were used as a control. Values are expressed as mean ± SEM (*n* = 9). * Significant (*p* < 0.0001) difference compared to all other groups. LPS (*E. coli* lipopolysaccharide), Extract (*C. sylvestris* ethanolic extract), F2 (*C. sylvestris* diterpene-concentrated fraction), Cas J (*C. sylvestris* clerodane diterpene casearin J).

Subsequently, the protein levels of TNF-α, IL-8, and MMP-1 in the cell supernatants were measured by ELISA. Corresponding to the expression levels, LPS led to a significant (*p* < 0.0001) increase in TNF-α, IL-8, and MMP-1, whereas *C. sylvestris* extract, F2, or Cas J could significantly (*p* < 0.0001) counteract this LPS-induced stimulation (Figure 4a–f).

### 3.2. Modulation of NO Levels by C. sylvestris Derivatives

**Figure 5:**
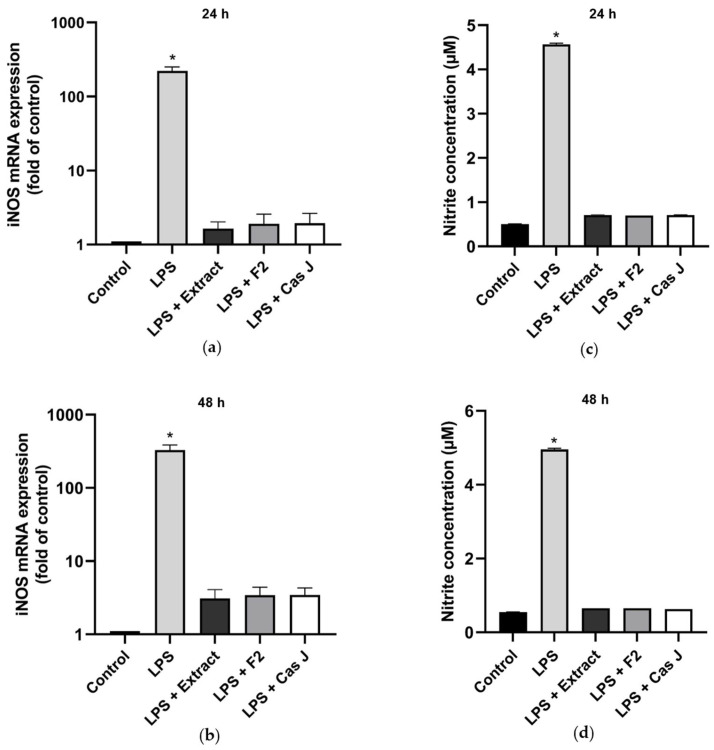
Regulation of *iNOS* gene expression (**a**,**b**) and NO production (**c**,**d**), as measured by nitrite concentration, in supernatants of gingival keratinocytes exposed to LPS in the presence and absence of *C. sylvestris* derivatives after 24 h and 48 h. Untreated cells were used as a control. Values are expressed as mean ± SEM (*n* = 9). Log10 scale (**a**,**b**). * Significant (*p* < 0.0001) difference compared to all other groups. LPS (*E. coli* lipopolysaccharide), Extract (*C. sylvestris* ethanolic extract), F2 (*C. sylvestris* diterpene-concentrated fraction), Cas J (*C. sylvestris* clerodane diterpene casearin J).

We then examined the effects of the tested compounds on the inducible nitric oxide synthase (*iNOS*) gene expression and nitric oxide (NO) concentration by real-time PCR and Griess reaction, respectively, after 24 h and 48 h. LPS significantly (*p* < 0.0001) enhanced the *iNOS* expression (Figure 5a,b, Table S1) and NO synthesis (Figure 5c,d) at 24 h and 48 h. Again, *C. sylvestris* extract, F2, or Cas J significantly (*p* < 0.0001) abrogated the stimulatory LPS effects (Figure 5a–d).

### 3.3. Effect of C. sylvestris Derivatives on NF-κB p65 Nuclear Translocation

**Figure 6:**
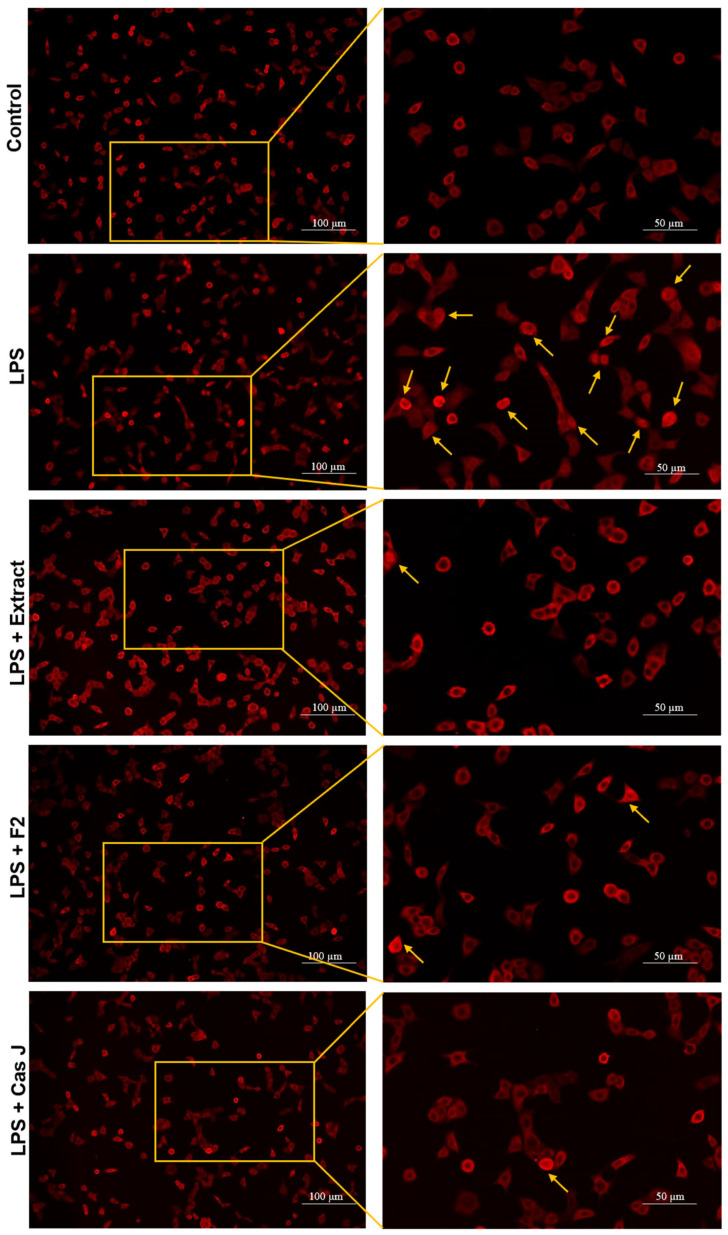
Regulation of the NF-κB signaling pathway in gingival keratinocytes exposed to LPS in the presence of *C. sylvestris* derivatives, as analyzed by immunofluorescence microscopy. Representative images of the groups from one experiment are shown. Yellow arrows indicate NF-κB nuclear translocation. Untreated cells were used as a control. LPS (*E. coli* lipopolysaccharide), Extract (*C. sylvestris* ethanolic extract), F2 (*C. sylvestris* diterpene-concentrated fraction), Cas J (*C. sylvestris* clerodane diterpene casearin J).

To elucidate the mechanisms by which *C. sylvestris* compounds could exert their regulatory effects, we investigated the activation of the nuclear factor-kappa B (NF-κB) signaling pathway in gingival keratinocytes exposed to LPS in the presence and absence of *C. sylvestris* derivatives. As shown in Figure 6, LPS resulted in nuclear translocation of NF-κB p65 in many cells at 60 min. The tested compounds inhibited the LPS-stimulated NF-κB p65 nuclear translocation at 60 min, as demonstrated by immunofluorescence microscopy.

### 3.4. Effects of LPS and C. sylvestris Derivatives on Cell Viability

**Figure 7:**
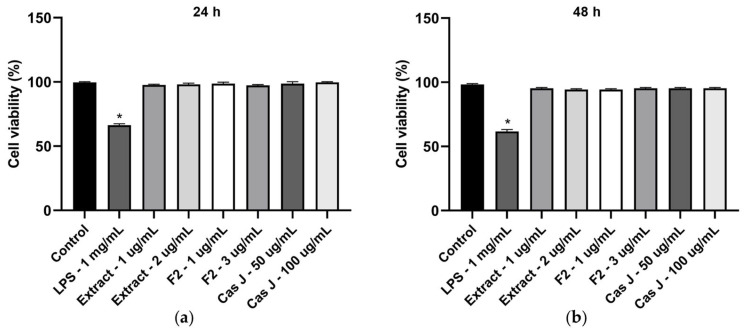
Cell viability of gingival keratinocytes exposed to LPS (1 µg/mL) in the presence and absence of *C. sylvestris* extract (1 µg/mL and 2 µg/mL), F2 (1 µg/mL and 3 µg/mL), and Cas J (50 µg/mL and 100 µg/mL) after 24 h (**a**) and 48 h (**b**). Untreated cells were used as a control. Values are expressed as mean ± SEM (*n* = 9). * Significant (*p* < 0.0001) difference compared to all other groups. LPS (*E. coli* lipopolysaccharide), Extract (*C. sylvestris* ethanolic extract), F2 (*C. sylvestris* diterpene-concentrated fraction), Cas J (*C. sylvestris* clerodane diterpene casearin J).

We also analyzed the cell viability of gingival keratinocytes exposed to LPS or different concentrations of *C. sylvestris* derivatives. Exposure to LPS resulted in a significant (*p* < 0.0001) reduction in cell viability compared to all other groups after 24 h and 48 h. However, cell viability was not compromised by *C. sylvestris* derivatives, regardless of the concentrations tested (Figure 7a,b).

### 3.5. Effect of C. sylvestris Derivatives on In Vitro Wound Healing and Cell Migration

**Figure 8:**
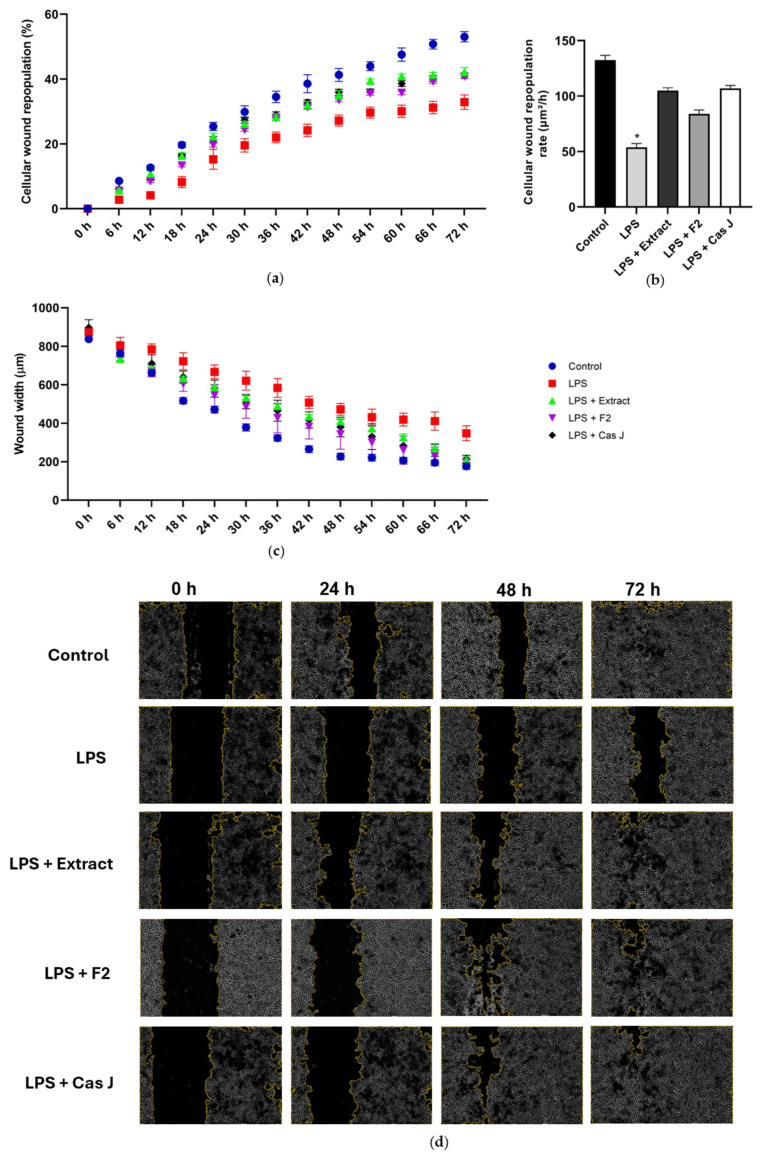
Percentage of cellular wound repopulation (**a**), wound width (μm) (**b**), and wound cell repopulation rate (μm^2^/h) (**c**) in gingival keratinocytes exposed to LPS in the presence and absence of *C. sylvestris* derivatives. Representative high-contrast brightfield images with 4× magnification captured using the Lionheart FX Automated Microscope (Agilent, Santa Clara, CA, USA) at 0 h, 24 h, 48 h, and 72 h for each group from one experiment (**d**). Untreated cells were used as a control. Values are expressed as mean ± SEM (*n* = 9). * Significant (*p* < 0.0001) difference compared to all other groups. LPS (*E. coli* lipopolysaccharide), Extract (*C. sylvestris* ethanolic extract), F2 (*C. sylvestris* diterpene-concentrated fraction), Cas J (*C. sylvestris* clerodane diterpene casearin J).

Finally, we studied the actions of *C. sylvestris* compounds on wound healing in vitro. The exposure of the wounded monolayers of gingival keratinocytes to LPS resulted in a reduced cellular wound repopulation, as shown in Figure 8a. In accordance, the cellular wound repopulation rate was significantly (*p* < 0.0001) decreased by LPS (Figure 8b). Furthermore, LPS inhibited the wound closure of 72 h, as assessed by the wound width (Figure 8c,d). However, the inhibitory effects of LPS on the cellular wound repopulation and its rate as well as the wound closure were counteracted by *C. sylvestris* extract, F2, and Cas J over 72 h (Figure 8a–d).

## 4. Discussion

This study provides novel in vitro evidence that *C. sylvestris* derivatives exert potent anti-inflammatory, antiproteolytic, and antioxidant effects on gingival keratinocytes exposed to *Escherichia coli* LPS. Furthermore, *C. sylvestris* compounds demonstrated wound healing potential without affecting cell viability, supporting their role as promising adjunctive agents for periodontal prevention and therapy.

*C. sylvestris* is a traditional Latin American medicinal plant widely used to treat inflammatory and ulcerative conditions [19]. Furthermore, *C. sylvestris* exhibits antimicrobial action against pathogens such as fungi and bacteria [23,24]. Its pharmacological effects are attributed to a rich phytochemical profile, including flavonoids and clerodane diterpenes, such as casearin J [25,26]. The search for herbal adjuvants is a growing area in oral and periodontal medicine [16,17,18]. Our results highlight the potential of *C. sylvestris* as a candidate for such use, especially due to its broad-spectrum anti-inflammatory and tissue-protective activities.

Periodontal disease involves the chronic production of inflammatory cytokines, such as TNF-α, IL-1β, IL-6, IL-8, and IL-17, which contribute to the destruction of soft and hard tissues [5,7,10]. In this study, gingival keratinocytes exposed to LPS, which is a component of the bacterial cell wall, significantly increased the aforementioned cytokines at the gene and protein levels, mimicking the pro-inflammatory microenvironment of gingival inflammation. Interestingly, when *C. sylvestris* derivatives were added to the cells simultaneously with LPS, there was a downregulation of these mediators, demonstrating a strong immunomodulatory potential. Additionally, we found that *IL-4*, an anti-inflammatory cytokine linked to the Th2 immune response, was significantly suppressed following exposure to LPS. IL-4 plays an important role in regulating inflammation, so its reduction may compromise immune regulatory mechanisms [5,7]. Interestingly, *C. sylvestris* treatment restored *IL-4* levels, suggesting a modulating effect that favors a more balanced, less inflammatory immune environment. In addition, the observed upregulation of *IL-10* by LPS, which is often associated with persistent inflammation, was normalized by *C. sylvestris* derivatives, indicating a balancing effect on immune homeostasis [19]. Furthermore, iNOS also plays a pro-inflammatory role in periodontal diseases. Elevated expression of iNOS and increased NO production have been measured in the gingival tissues, gingival crevicular fluid, and saliva of patients with periodontitis and gingivitis, and are often associated with disease severity and tissue destruction [9]. In the present study, LPS also induced the upregulation of *iNOS* and increased NO production, suggesting the induction of nitrosative stress, which plays a key role in tissue degradation [9]. Again, all tested *C. sylvestris* compounds reduced *iNOS* expression and NO accumulation, confirming their anti-inflammatory and antioxidant properties. These results are in agreement with previous studies describing *C. sylvestris* as an inhibitor of NO production and oxidative mediator in models of inflammation and tissue injury [19,28]. These data suggest that *C. sylvestris* could be a potential adjuvant for prevention and therapy of periodontal diseases by its anti-inflammatory and antioxidant effects.

When inflammation persists, intensifies, and endures over time, it can lead to soft tissue degradation and bone resorption. For example, MMP overexpression caused by inflammation results in collagen degradation and, thereby, periodontal attachment loss [7,10]. In the present study, LPS significantly increased the expression of *MMP-1* and *MMP-13*, while all *C. sylvestris* derivatives abrogated these effects. This antiproteolytic activity of *C. sylvestris* has also been demonstrated in other inflammatory conditions and may be attributed to its ability to suppress NF-κB and possibly mitogen-activated protein kinase (MAPK) signaling [19,28,29]. These data suggest that *C. sylvestris* could be a potential adjuvant in the prevention and therapy of periodontal diseases due to its antiproteolytic effects.

LPS can induce inflammation through toll-like receptor 4 (TLR4) activation, leading to nuclear translocation of NF-κB p65 [9,10]. Immunofluorescence microscopy confirmed this stimulatory effect of LPS on several cells in the present study. However, *C. sylvestris* inhibited NF-κB p65 nuclear translocation caused by LPS, suggesting a key mechanism for its anti-inflammatory bioactivity. This inhibition of NF-κB aligns with anti-inflammatory mechanisms described in other herbal medicines, such as curcumin, green tea, and passion fruit bagasse extract [16,17,18]. Other pathways might also be involved in the modulatory effects of *C. sylvestris*. Our data suggest that the anti-inflammatory and antiproteolytic actions are at least partially mediated through the NF-κB pathway.

We also studied the effects of *C. sylvestris* in an in vitro wound healing assay. Wound healing was significantly impaired by LPS, as demonstrated by delayed cellular wound repopulation and persistent wound width. Interestingly, *C. sylvestris* derivatives could counteract this negative influence of LPS by improving all wound healing parameters. It is important to highlight that cell viability was reduced by LPS, but remained unaffected by *C. sylvestris*, confirming the safety of its derivatives even at higher concentrations. Our findings are consistent with those of a previous study on the cytotoxicity of *C. sylvestris* [19]. The wound healing effects of *C. sylvestris* parallel those of other botanicals such as *Aloe vera*, *Calendula officinalis*, and *Chamomilla recutita*, which contain antioxidant flavonoids that promote wound healing [30,31,32]. So far, *C. sylvestris* has been used in the treatment of cutaneous ulcers and wounds [19,33]. Our study suggests that *C. sylvestris* could also promote oral wound healing.

In the search for effective natural agents to control oral inflammation, several other plant-derived compounds and biopolymers have been investigated. Chitosan, for instance, has shown antimicrobial and wound healing-promoting properties and has been used as a coating material for dental applications [34,35]. Propolis, a resinous substance produced by bees, is well recognized for its anti-inflammatory and antioxidant properties in periodontal therapy [36,37]. Furthermore, cranberry extracts, rich in proanthocyanidins, have demonstrated the ability to inhibit bacterial adhesion and modulate host inflammatory responses [38]. In the present study, *C. sylvestris* derivatives significantly reduced the production of inflammatory mediators (*TNF-α*, *IL-8*, *iNOS*), decreased proteolytic activity, and promoted wound healing in gingival keratinocytes, reinforcing their potential as a novel adjuvant therapy for the control of periodontal inflammation.

The present investigation has also some limitations. In our study, we focused on human gingival keratinocytes to investigate possible regulatory effects of *C. sylvestris*. These cells are located at the interface between bacteria and host serving as the first line of defense of the oral mucosa against periodontal pathogens and directly mediating the initial signaling of the inflammatory response [39,40]. Furthermore, these cells play a central role in the production of pro-inflammatory and chemotactic cytokines shortly after the recognition of microorganisms or their products making them an important model for the investigation of epithelial reactions in gingivitis [40,41]. However, periodontal tissues are composed of a variety of different cell types that interact dynamically during inflammation and healing. Therefore, future studies should also explore the response of other periodontal cell types, such as gingival fibroblasts, periodontal ligament fibroblasts, and bone cells. In addition, the inflammatory stimulus in our study was induced with *E. coli* LPS. Our data show that LPS from *E. coli* exerted strong pro-inflammatory effects on gingival keratinocytes. We used *E. coli* LPS in our study because it is a standardized, purified form of LPS that is frequently used in research, making it easy to compare with other studies [42,43,44,45]. Furthermore, studies have shown that the effects of *E. coli* LPS are similar to those of periodontopathogens, such as *P. gingivalis*, with regard to the activation of inflammatory pathways, such as NF-κB, and the expression of pro-inflammatory cytokines [44,46]. However, due to the polymicrobial nature and structural complexity of periodontal biofilms, future studies should also incorporate other bacteria and bacterial biofilms to better mimic the complex environment of periodontal diseases. Although the study demonstrated significant biological effects of *C. sylvestris* derivatives, the extract was not fully characterized phytochemically, leaving other potentially active constituents unidentified. Finally, as this is an in vitro study, pre-clinical in vivo and clinical trials are needed to validate the therapeutic potential, safety, and mechanisms of action of *C. sylvestris* in the context of periodontal diseases.

## 5. Conclusions

This in vitro study provides novel evidence that *C. sylvestris* leaf derivatives, such as the crude ethanolic extract, a concentrated diterpene fraction, and the diterpene casearin J isolated from clerodane, exert significant anti-inflammatory, antiproteolytic, and antioxidant effects on gingival keratinocytes exposed to *E. coli* LPS. Furthermore, the *C. sylvestris* derivatives also promoted wound healing by increasing cell migration and repopulation in vitro, without compromising cell viability even at higher concentrations, thus confirming their biological safety. These findings support the potential use of *C. sylvestris* as an adjunctive agent for periodontal prevention and therapy. However, pre-clinical in vivo and clinical trials are needed to validate the efficacy, safety, and mechanisms of action of *C. sylvestris*.

## Data Availability

Data used in the present study are available from the corresponding author on reasonable request.

## Supplementary Materials

The following supporting information can be downloaded at: https://www.mdpi.com/article/10.3390/antiox14080901/s1, Table S1: Inhibition of LPS-induced gene expression (%) by extract, F2 and cas J in gingival keratinocytes at 24 h and 48 h. Extract (*C. sylvestris* ethanolic extract), F2 (*C. sylvestris* diterpene-concentrated fraction), Cas J (*C. sylvestris* clerodane diterpene casearin J).

## Abbreviations

The following abbreviations are used in this manuscript:

IL-1β	Interleukin-1 beta
TNF-α	Tumor necrosis factor-alpha
IL-6	Interleukin-6
IL-23	Interleukin-23
IL-17	Interleukin-17
*C. sylvestris*	*Casearia sylvestris*
LPS	Lipopolysaccharide
Extract	*Casearia sylvestris* ethanolic extract
F2	*Casearia sylvestris* diterpene-concentrated fraction
Cas J	*Casearia sylvestris* clerodane diterpene casearin J
IL-8	Interleukin-8
IL-4	Interleukin-4
IL-10	Interleukin-10
MMP-1	Matrix metalloproteinase-1
MMP-13	Matrix metalloproteinase-13
iNOS	Inducible nitric oxide synthase
NO	Nitric oxide
NF-κB	Nuclear factor-kappa B
*E. coli*	*Escherichia coli*
Th2	T helper 2 cells
MAPK	Mitogen-activated protein kinase
TLR4	Toll-like receptor 4
*P. gingivalis*	*Porphyromonas gingivalis*
TIGKs	Telomerase-immortalized gingival keratinocytes
ATCC	American type culture collection
HPLC	High performance liquid chromatography
PDA	Photodiode array
UV	Ultraviolet
PCR	Polymerase chain reaction
RNA	Ribonucleic acid
cDNA	Complementary deoxyribonucleic acid
ELISA	Enzyme-linked immunosorbent assay
SEM	Standard error of the mean

## References

[1] Squier C.A., Kremer M.J. (2001). Biology of oral mucosa and esophagus. J. Natl. Cancer Inst. Monogr..

[2] Nanci A., Bosshardt D.D. (2006). Structure of periodontal tissues in health and disease. Periodontology 2000.

[3] Trombelli L., Farina R., Silva C.O., Tatakis D.N. (2018). Plaque-induced gingivitis: Case definition and diagnostic considerations. J. Periodontol..

[4] Neurath N., Kesting M. (2024). Cytokines in gingivitis and periodontitis: From pathogenesis to therapeutic targets. Front. Immunol..

[5] Leite F.R.M., Nascimento G.G., Møller H.J., Belibasakis G.N., Bostanci N., Smith P.C., López R. (2022). Cytokine profiles and the dynamic of gingivitis development in humans. J. Clin. Periodontol..

[6] Kolte A.P., Kolte R.A., Bawankar P.V., Bajaj V.A. (2023). Assessment and correlation of the influence of non-surgical periodontal therapy on serum lipid profile and cytokines in patients with stage III periodontitis. Int. J. Dent. Hyg..

[7] Blanco-Pintos T., Regueira-Iglesias A., Balsa-Castro C., Tomás I. (2022). Update on the role of cytokines as oral biomarkers in the diagnosis of periodontitis. Advances in Experimental Medicine and Biology.

[8] Alarcón-Sánchez M.A., Guerrero-Velázquez C., Becerra-Ruiz J.S., Rodríguez-Montaño R., Avetisyan A., Heboyan A. (2024). IL-23/IL-17 axis levels in gingival crevicular fluid of subjects with periodontal disease: A systematic review. BMC Oral Health.

[9] Chapple I.L., Matthews J.B. (2007). The role of reactive oxygen and antioxidant species in periodontal tissue destruction. Periodontology 2000.

[10] Eisenbarth S.C., Piggott D.A., Huleatt J.W., Visintin I., Herrick C.A., Bottomly K. (2002). Lipopolysaccharide-enhanced, toll-like receptor 4-dependent T helper cell type 2 responses to inhaled antigen. J. Exp. Med..

[11] Lang N.P., Bartold P.M. (2018). Periodontal health. J. Clin. Periodontol..

[12] Sanz M., Beighton D., Curtis M.A., Cury J.A., Dige I., Dommisch H., Ellwood R., Giacaman R.A., Herrera D., Herzberg M.C. (2017). Role of microbial biofilms in the maintenance of oral health and in the development of dental caries and periodontal diseases. Consensus report of group 1 of the Joint EFP/ORCA workshop on the boundaries between caries and periodontal disease. J. Clin. Periodontol..

[13] Sanz M., Herrera D., Kebschull M., Chapple I., Jepsen S., Beglundh T., Sculean A., Tonetti M.S., On behalf of the EFP Workshop Participants and Methodological Consultants (2020). Treatment of stage I–III periodontitis—The EFP S3 level clinical practice guideline. J. Clin. Periodontol..

[14] Paradowska-Stolarz A., Wieckiewicz M., Owczarek A., Wezgowiec J. (2021). Natural polymers for the maintenance of oral health: Review of recent advances and perspectives. Int. J. Mol. Sci..

[15] Shetty S., Thomas B., Shetty V., Bhandary R., Shetty R.M. (2013). An in-vitro evaluation of the efficacy of garlic extract as an antimicrobial agent on periodontal pathogens: A microbiological study. Ayu (Int. Q. J. Res. Ayurveda).

[16] Mathur A., Gopalakrishnan D., Mehta V., Rizwan S.A., Shetiya S.H., Bagwe S. (2018). Efficacy of green tea-based mouthwashes on dental plaque and gingival inflammation: A systematic review and meta-analysis. Indian J. Dent. Res..

[17] Forouzanfar F., Forouzanfar A., Sathyapalan T., Orafai H.M., Sahebkar A. (2020). Curcumin for the management of periodontal diseases: A review. Curr. Pharm. Des..

[18] Nogueira A.V.B., Faria L.V., Lopes M.E.S., Viganó J., Martínez J., Eick S., Cirelli J.A., Deschner J. (2025). Anti-inflammatory properties of yellow passion fruit bagasse extract and its potential role in periodontal wound healing in vitro. Biomedicines.

[19] Pierri E.G., Castro R.C., Vizioli E.O., Ferreira C.M.R., Cavalheiro A.J., Tininis A.G., Chin C.M., Santos A.G. (2017). Anti-inflammatory action of ethanolic extract and clerodane diterpenes from *Casearia sylvestris*. Rev. Bras. Farmacogn..

[20] Ferreira P.M., Militão G.C., Lima D.J., Costa N.D., da Conceição Machado K., Santos A.G., Cavalheiro A.J., da Silva Bolzani V., Silva D.H., Pessoa C. (2014). Morphological and biochemical alterations activated by antitumor clerodane diterpenes. Chem. Biol. Interact..

[21] Spósito L., Oda F.B., Vieira J.H., Carvalho F.A., Dos Santos Ramos M.A., de Castro R.C., Crevelin E.J., Crotti A.E.M., Santos A.G., da Silva P.B. (2019). In vitro and in vivo anti-Helicobacter pylori activity of *Casearia sylvestris* leaf derivatives. J. Ethnopharmacol..

[22] Esteves I., Souza I.R., Rodrigues M., Cardoso L.G., Santos L.S., Sertie J.A., Perazzo F.F., Lima L.M., Schneedorf J.M., Bastos J.K. (2005). Gastric antiulcer and anti-inflammatory activities of the essential oil from *Casearia sylvestris* Sw. J. Ethnopharmacol..

[23] Ribeiro S.M., Bueno P.C.P., Cavalheiro A.J., Klein M.I. (2023). Effect of extracts, fractions, and isolated molecules of *Casearia sylvestris* to control *Streptococcus mutans* cariogenic biofilm. Antibiotics.

[24] Ribeiro S.M., Fratucelli É.D.O., Bueno P.C.P., de Castro M.K.V., Francisco A.A., Cavalheiro A.J., Klein M.I. (2019). Antimicrobial and antibiofilm activities of *Casearia sylvestris* extracts from distinct Brazilian biomes against *Streptococcus mutans* and *Candida albicans*. BMC Complement. Altern. Med..

[25] Alexandre Carvalho F., Valadares de Moraes N., Eduardo Miller Crotti A., José Crevelin E., Gonzaga Dos Santos A. (2023). *Casearia* essential oil: An updated review on the chemistry and pharmacological activities. Chem. Biodivers..

[26] Lago J.H., Toledo-Arruda A.C., Mernak M., Barrosa K.H., Martins M.A., Tibério I.F., Prado C.M. (2014). Structure-activity association of flavonoids in lung diseases. Molecules.

[27] Carvalho F.A., Uchina H.S., Borges F.A., Oyafuso M.H., Herculano R.D., Gremião M.P.D., Santos A.G. (2018). Natural membranes of Hevea brasiliensis latex as delivery system for *Casearia sylvestris* leaf components. Rev. Bras. Farmacogn..

[28] Albano M.N., da Silveira M.R., Danielski L.G., Florentino D., Petronilho F., Piovezan A.P. (2013). Anti-inflammatory and antioxidant properties of hydroalcoholic crude extract from *Casearia sylvestris* Sw. (Salicaceae). J. Ethnopharmacol..

[29] Ferreira B.A., Silva R.F., de Moura F.B.R., Narduchi C.T., Deconte S.R., Sartorelli P., Tomiosso T.C., Lago J.H.G., Araújo F.A. (2022). α-zingiberene, a sesquiterpene from essential oil from leaves of *Casearia sylvestris*, suppresses inflammatory angiogenesis and stimulates collagen deposition in subcutaneous implants in mice. Nat. Prod. Res..

[30] Teplicki E., Ma Q., Castillo D.E., Zarei M., Hustad A.P., Chen J., Li J. (2018). The effects of Aloe vera on wound healing in cell proliferation, migration, and viability. Wounds.

[31] Tanideh N., Tavakoli P., Saghiri M.A., Garcia-Godoy F., Amanat D., Tadbir A.A., Samani S.M., Tamadon A. (2013). Healing acceleration in hamsters of oral mucositis induced by 5-fluorouracil with topical Calendula officinalis. Oral Surg. Oral Med. Oral Pathol. Oral Radiol..

[32] Martins M.D., Marques M.M., Bussadori S.K., Martins M.A., Pavesi V.C., Mesquita-Ferrari R.A., Fernandes K.P. (2009). Comparative analysis between Chamomilla recutita and corticosteroids in wound healing. An in vitro and in vivo study. Phytother. Res..

[33] de Oliveira B.M.M., Serpa P.Z., da Costa Zanatta M.E., Aires B.A., Steffler A.M., Somensi L.B., Cury B.J., Dos Santos A.C., Venzon L., Boeing T. (2022). Gastroprotective and gastric healing effects of the aqueous extract of *Casearia sylvestris* in rodents: Ultrasound, histological and biochemical analyzes. J. Ethnopharmacol..

[34] Thangavelu A., Stelin K.S., Vannala V., Mahabob N., Hayyan F.M.B., Sundaram R. (2021). An overview of chitosan and its role in periodontics. J. Pharm. Bioallied Sci..

[35] Pavez L., Tobar N., Chacón C., Arancibia R., Martínez C., Tapia C., Pastor A., González M., Martínez J., Smith P.C. (2018). Chitosan-triclosan particles modulate inflammatory signaling in gingival fibroblasts. J. Periodontal Res..

[36] Luque-Bracho A., Rosales Y., Vergara-Buenaventura A. (2023). The benefits of propolis in periodontal therapy: A scoping review of preclinical and clinical studies. J. Ethnopharmacol..

[37] López-Valverde N., Pardal-Peláez B., López-Valverde A., Flores-Fraile J., Herrero-Hernández S., Macedo-de-Sousa B., Herrero-Payo J., Ramírez J.M. (2021). Effectiveness of propolis in the treatment of periodontal disease: Updated systematic review with meta-analysis. Antioxidants.

[38] Olczak-Kowalczyk D., Turska-Szybka A., Twetman S., Gozdowski D., Piekoszewska-Ziętek P., Góra J., Wróblewska M. (2025). Effect of tablets containing a paraprobiotic strain and the cranberry extract on caries incidence in preschool children: A randomized controlled trial. Dent. Med. Probl..

[39] Groeger S., Meyle J. (2019). Oral mucosal epithelial cells. Front. Immunol..

[40] Overmiller A.M., Sawaya A.P., Hope E.D., Morasso M.I. (2022). Intrinsic networks regulating tissue repair: Comparative studies of oral and skin wound healing. Cold Spring Harb. Perspect. Biol..

[41] Griffin M.F., Fahy E.J., King M., Guardino N., Chen K., Abbas D.B., Lavin C.V., Diaz Deleon N.M., Lorenz H.P., Longaker M.T. (2022). Understanding scarring in the oral mucosa. Adv. Wound Care.

[42] Yang X., Cai X., Lin J., Zheng Y., Liao Z., Lin W., He X., Zhang Y., Ren X., Liu C. (2024). *E. coli* LPS-induced calcium signaling regulates the expression of hypoxia-inducible factor 1α in periodontal ligament fibroblasts in a non-hypoxia-dependent manner. Int. Immunopharmacol..

[43] Chen H., Peng L., Wang Z., He Y., Zhang X. (2024). Influence of METTL3 knockdown on PDLSC osteogenesis in E. coli LPS-induced inflammation. Oral Dis..

[44] Behm C., Blufstein A., Abhari S.Y., Koch C., Gahn J., Schäffer C., Moritz A., Rausch-Fan X., Andrukhov O. (2020). Response of human mesenchymal stromal cells from periodontal tissue to LPS depends on the purity but not on the LPS source. Mediat. Inflamm..

[45] Wang Y., Gong J., Zeng H., Liu R., Jin B., Chen L., Wang Q. (2016). Lipopolysaccharide activates the unfolded protein response in human periodontal ligament fibroblasts. J. Periodontol..

[46] Nativel B., Couret D., Giraud P., Meilhac O., d’Hellencourt C.L., Viranaïcken W., Da Silva C.R. (2017). Porphyromonas gingivalis lipopolysaccharides act exclusively through TLR4 with a resilience between mouse and human. Sci. Rep..

