# Supplementary material for: Anti-Inflammatory, Antioxidant, and Reparative Effects of Casearia sylvestris Leaf Derivatives on Periodontium In Vitro
Source: Antioxidants (Basel). 2025 Jul 23;14(8):901. doi: 10.3390/antiox14080901 (PMC12382619; doi:10.3390/antiox14080901)
Supplement: Supplementary file 1 [file antioxidants-14-00901-s001.zip › antioxidants-3698920-supplementary.pdf]

Supplementary File

Table S1. Inhibition of LPS-induced gene expression (%) by extract, F2 and cas J in gingival keratinocytes at 24 h and 48 h. Extract (*C. sylvestris* ethanolic extract), F2 (*C. sylvestris* diterpene-concentrated fraction), Cas J (*C. sylvestris* clerodane diterpene casearin J).

	Extract		F2		Cas J	
	**24 h**	**48 h**	**24 h**	**48 h**	**24 h**	**48 h**
**TNF-a**	99.72	99.72	99.74	99.74	99.74	99.75
**IL-1β**	100.00	100.00	100.00	100.00	100.00	100.00
**IL-4**	100.00	100.00	100.00	100.00	100.00	100.00
**IL-6**	99.27	99.42	99.46	99.49	99.48	99.50
**IL-8**	98.97	98.79	98.99	98.87	98.98	98.88
**IL-10**	99.63	99.58	99.55	99.54	99.73	99.73
**IL-17**	83.13	80.17	84.48	83.91	82.21	81.36
**iNOS**	99.19	99.20	99.06	99.11	99.04	99.11
**MMP-1**	91.01	90.33	93.38	93.34	88.94	88.36
**MMP-13**	99.02	98.80	99.17	98.96	99.29	98.97
